# Inhibition of STAT3 in tubular epithelial cells prevents kidney fibrosis and nephropathy in STZ-induced diabetic mice

**DOI:** 10.1038/s41419-019-2085-0

**Published:** 2019-11-07

**Authors:** Chao Zheng, Lan Huang, Wu Luo, Weihui Yu, Xueting Hu, Xinfu Guan, Yan Cai, Chunpeng Zou, Haimin Yin, Zheng Xu, Guang Liang, Yi Wang

**Affiliations:** 1 Chemical Biology Research Center, School of Pharmaceutical Science, Wenzhou Medical University, Wenzhou, Zhejiang 325035 China; 2 Department of Endocrinology, the Second Affiliated Hospital, Wenzhou Medical University, Wenzhou, Zhejiang 325000 China; 3 The Affiliated Cangnan Hospital, Wenzhou Medical University, Wenzhou, Zhejiang 325400 China; 4 Department of Endocrinology, the First Affiliated Hospital, Wenzhou Medical University, Wenzhou, Zhejiang 325035 China; 5 Department of Ultrasonography, the Second Affiliated Hospital, Wenzhou Medical University, 325000 Wenzhou, Zhejiang China

**Keywords:** Diabetes complications, Diabetes complications

## Abstract

Recent evidences indicate that signal transducer and activator of transcription 3 (STAT3) is one of the crucial signaling pathways in the progression of diabetic nephropathy (DN). Here, we investigated the hypothesis that pharmacological blockade of STAT3 limits the progression of DN. Treatment with selective STAT3 inhibitor, S3I-201 for 16 weeks significantly attenuated kidney injuries in streptozotocin (STZ) induced diabetic mice, associated with downregulated expression of TGF-β1, ACE/AT1, and VEGF in diabetic mouse kidneys. Similar results were confirmed using genetic knockdown of STAT3 in mouse kidneys by injections of AAV2 expressing STAT3 shRNA in diabetic mouse. Further, STAT3 localization in kidney tissue was evaluated using immunofluorescent double-staining analysis, which indicated that STAT3 expression was mainly in the tubular epithelial cells. As expected, in renal tubular epithelial NRK-52E cells, high glucose (HG)-induced overexpression of TGF-β1, ACE/AT1, and VEGF were abrogated by S3I-201 pretreatment, as well as by genetic knockdown of STAT3 using specific siRNA sequence. This study found that renal tubular epithelial cells contributed to STAT3-mediated progression of DN and provided the first evidence that pharmacological inhibition of STAT3 attenuates DN.

## Introduction

Diabetic nephropathy (DN) is the most common cause of end stage renal disease and kidney failure in many countries today, with about 40% of patients with either type 1 or type 2 diabetes developing DN^1^. With increasing incidences of diabetes, there is much need to understand the pathogenesis of DN in order to prevent and treat this devastating disease^1,2^. Over the years, the pathophysiological mechanisms of DN have been intensely investigated and are currently recognized to be multifactorial, with derangements in metabolism, hemodynamics, inflammation, and autophagy, leading to dysregulated signaling cascades including Janus kinase/signal transducer and activators of transcription (JAK/STAT), protein kinase C, and nuclear factor-κB (NF-κB)^3^.

JAK/STAT is a crucial signaling cascade in the pathogenesis of DN^4–6^. It is important in the regulation of fundamental cellular processes such as growth, differentiation, survival, and immunity. Its aberrant activity is also linked to carcinogenesis and tumor progression^7,8^, and has been of great interest as a therapeutic target. Evidence indicates that activation of the JAK/STAT pathway in renal glomerular mesangial cells stimulates excessive cell proliferation as well as enhanced production of TGF-β1, collagen IV, and fibronectin, contributing to the glomerulosclerosis in DN^9,10^. These findings are further corroborated with detection of higher levels of mRNA expression of JAKs 1,2,3 and STATs 1,3 in glomeruli and tubule-interstitial samples obtained from human subjects with diabetic kidney disease compare with that in normal subjects^11^. Moreover, streptozotocin(STZ)-induced diabetes of transgenic mice with reduced STAT3 activity develop less proteinuria, glomerular cell proliferation, and fibrotic activities than normal mice^12^. In addition, activated STAT3 in tubular cells has been reported to be important in chronic kidney disease^13^. In general, this current evidence supports a key role of STAT3 in the pathogenesis of DN. However, it is unknown if pharmacological inhibition of STAT3 by a small-molecule inhibitor is able to be a therapeutic strategy for DN.

In addition, our understanding of the STAT pathway in the pathogenesis of DN remains limited to mostly data obtained from mesangial cells, with little to no information on the contribution of STAT signaling by other cells in the kidney. In contrast, experimental models of acute kidney injury (i.e., ischemia/reperfusion injury, HIV) implicate pathogenic involvement of the STAT pathway in several renal cell types (i.e., podocytes, fibroblasts glomerular endothelium) as well as non-renal cells (i.e., renal tubular epithelium, immune cells)^4,14^. For example, experimental evidence obtained from a murine kidney injury model using unilateral ureteral obstruction links STAT3 with increased deposition of extracellular matrix proteins, thereby driving the progression of renal fibrosis^15^. Moreover, in this experimental model of kidney injury, STAT3 is found to be localized primarily in renal tubular epithelial cells of collecting ducts in normal and obstructed kidneys and interstitial cells in obstructed kidneys^16^. Findings from these reports indicate that several kidney cell populations appear to regulate disease processes through STAT3 signaling, at least in acute kidney injury. At present, the involvement of STAT3 signaling in the pathogenesis of DN by kidney cell populations other than mesangial cells remains unclear.

Therefore, we investigated the hypothesis that blockade of STAT3 limits the pro-fibrotic activities driving the progression of DN in a mouse of model of STZ-induced DM as well as in a high glucose (HG) in vitro model of renal tubular epithelial cells. The small molecule selective STAT3 inhibitor, S3I-201, which inhibits STAT3 DNA-binding and STAT3 phosphorylation^7^, was used to determine the efficiency of STAT3 inhibition in preventing DN in the in vivo and in vitro experimental models. The results provide the first evidence of the protective effect of pharmacological inhibition of STAT3 by S3I-201 against the progression of DN. Moreover, renal tubular epithelial cells contributed to the STAT3 pathogenic mechanism of DN.

## Materials and methods

### Reagents, cell culture, and treatment

S3I-201 was purchased from Selleck Chemicals (Cat no. S1155; Shanghai, China). S3I-201 was dissolved in DMSO (stock concentration: 10 mM) for in vitro experiments and in castor oil for in vivo experiments. Rat renal tubular epithelial cell line (NRK-52E) was obtained from the Shanghai Institute of Biochemistry and Cell Biology (Shanghai, China) and cultured in DMEM medium (Gibco, Eggenstein, Germany) containing 5.5 mM of D-glucose (low glucose, LG) supplemented with 10% FBS (Gibco), 100 U/ml of penicillin, and 100 mg/ml of streptomycin in a humidified atmosphere of 5% CO_2_ at 37 °C. Before treatment, NRK-52E cells were cultured in 60-mm plates for overnight. In the high glucose-treated group (HG), cells were incubated with a DMEM medium containing 33 mM of glucose. The cell treatments were described in the corresponding figure legends.

### Animal experiments

Male C57BL/6 mice were obtained from the Animal Center of Wenzhou Medical University. All animal procedures were approved by the Ethics Committee of Wenzhou Medical University Animal Policy and Welfare Committee. Mice were housed in an environmentally controlled room at 22 ± 2.0 °C and 50% ± 5% humidity with a 12-h:12-h light/dark cycle and fed food and water ad libitum. All animal experiments were performed and analyzed by blinded experimenters. Treatment groups were assigned in a randomized fashion.

#### Pharmacological inhibition of STAT3 in diabetic mice

The randomized animal division results in 3 treatment groups in total. (i) Control group with normal blood glucose (Ctrl group, *n* = 7), (ii) STZ-induced diabetic mice (T1DM group, *n* = 7), (iii) STZ-induced diabetic mice treated with S3I-201 (T1DM+S3I-201 group, *n* = 7). Diabetes mellitus was induced in mice at 6–8 weeks old, weighing 18–20 g by intraperitoneal (IP) injection of streptozotocin (STZ, Sigma Chemicals, St. Louis, MO) at the dosage of 50 mg/kg dissolved in 100 mM citrate buffer (pH 4.5) for five consecutive days. Seven control mice received the same volume of citrate buffer. After two weeks, blood was collected by mandibular vein puncture for measures of glucose using a Glucometer. Fourteen mice with a fasting-blood glucose > 12 mmol/L were considered diabetic and were used for the further study. Treatment with S3I-201 was initiated after the establishment of frank type 1 DM. Diabetic mice were treated with S3I-201 (2.5 mg/kg) by IP injection three times a week for 16 weeks (*n* = 7 in each group). Mice in Control and T1DM groups were intraperitoneally administrated with the vehicle in the same schedule. At the indicated time points (0–18th week), blood glucose was determined and body weight recorded once every week. Eighteen weeks after STZ treatment, the final body weight and kidney weight were measured, and blood sample collected before the mice were killed under anesthesia.

#### Knockdown of STAT3 in diabetic mice

AAV2/2-U6-shSTAT3 recombinant (titer 2.6 × 10^12^ GC/ml) and AAV2/2-U6-NC recombinant (titer 6.4 × 10^12^ GC/ml) were purchased from Genechem Company (Shanghai, China). The following sequences for the shSTAT3 and the negative control were used: shSTAT3: 5′-aattcgCAGGTATCTTGAGAAGCCAATGGA AttcaagagaTTCCATTGGCTTCTCAAGATACCTGttttttg-3′; NC: 5'-aattcgTTCTCCGAAC GTGTCACGTAAttcaagagaTTACGTGACACGTTCGGAGAAttttttg-3′. To knockdown STAT3 in mouse kidney, we injected AAV2/2 expressing STAT3 shRNA by tail vein 2 weeks before STZ injection. The control groups received the same volume of AAV2/2 vehicle expressing negative control sequence (AAV-NC). Eighteen-weeks-old C57BL/6 mice (*n* = 28) were randomly allocated initially into two big groups (*n* = 14 per group). The mice in AAV2/2-shSTAT3 group were further divided such that seven mice were randomized to receive intraperitoneal (IP) injection of streptozotocin at the dosage of 50 mg/kg dissolved in 100 mM citrate buffer (pH 4.5) for three consecutive days and 7 to receive an equal volume of vehicle. Similarly, the AAV2/2-NC control animals were randomized either to receive STZ injections (*n* = 7) or vehicle (*n* = 7), resulting in four treatment groups in total: (i) AAV2/2-NC-treated control mice that received phosphate buffered saline (PBS) (AAV2/2-NC group, *n* = 7); (ii) AAV2/2-NC-treated mice with STZ (AAV2/2-NC+STZ group, *n* = 7); (iii) AAV2/2-shSTAT3-treated mice that received PBS (AAV2/2-shSTAT3, *n* = 7); (iv) AAV2/2-shSTAT3-treated mice with STZ (AAV2/2-shSTAT3+Ang II, *n* = 7). At the indicated time points (0–17th week), blood glucose was determined and body weight recorded once every week. Seventeen weeks after STZ treatment, the final body weight and kidney weight were measured, and blood sample collected before the mice were killed under anesthesia.

The collected blood samples were centrifuged at 4 °C at 3000 rpm for 10 min to collect serum. Kidney tissues were either fixed in 4% paraformaldehyde for pathological analysis or flash-frozen in liquid nitrogen for gene and protein expression analysis.

### Reverse transcription and real-time quantitative PCR

Total RNA was isolated from cells and tissues using TRIZOL (Invitrogen, Carlsbad, CA) according to the manufacturer's instructions. Reverse transcription and quantitative PCR were performed using M-MLV Platinum RT-qPCR Kit (Invitrogen, Carlsbad, CA). Real-time qPCR was carried out using the Eppendorf Real-plex 4 instrument (Eppendorf, Hamburg, Germany). Primers for genes were synthesized by Invitrogen (Invitrogen, Shanghai, China) and shown in Supplementary Table S1.

### Western blot analysis

Cell and tissue lysate homogenates were prepared. In every western blot analysis, the same amount of total protein from each group was separated by 8–12% SDS-PAGE and electro-transferred onto a nitrocellulose membrane (Bio-Rad Laboratory, Hercules, CA). The membrane was blocked for 1 h at room temperature in TBST (Tris-buffered saline with 0.05% Tween 20, pH 7.4) plus 5% non-fat milk, and incubated with primary antibodies overnight at 4 °C. Antibodies against p-STAT3 and STAT3 were obtained from CST (Cat. #9145 and #4905S, respectively), antibodies against ACE (Cat. #SC-2908), AT1 (Cat. #SC-2225), TGF-β (Cat. #SC-146), and VEGF (Cat. #SC-152) were purchased from Santa Cruz Technology (Santa Cruz, CA). Antibody for GAPDH was purchased from Abcam BioTech (Cat. #AB-P-R001). After three washes with TBST, membranes were incubated with the appropriate secondary antibody (Santa Cruz, CA; 1:3000) for 1–2 h at room temperature. The signals were visualized using enhanced chemiluminescence reagents (Bio-Rad, Hercules, CA). Band intensities were quantified using Image J software (NIH, Bethesda, MD).

### Immunohistochemical determination

The fixed kidney tissues were cut into segments (~2–3 mm in length) for dehydration in a graded alcohol series, cleared with xylene, embedded in paraffin, and sectioned at 5 µm thickness. For immunohistological preparations, paraffin sections were dewaxed, rehydrated in graded alcohol series, subjected to antigen retrieval in 0.01 mol/L citrate buffer (pH 6.0) for 3 min at 98 °C, and placed in 3% hydrogen peroxide in methanol for 30 min at room temperature. After blocking with 5% BSA, these sections were then incubated with primary antibodies against p-STAT3 (Tyr-705) and STAT3 (CST, USA) at 1:200 dilution overnight at 4 °C, followed by incubation with the appropriate secondary antibodies (1:200 Santa Cruz, CA, USA). The reaction was visualized with DAB solution. After counterstaining with hematoxylin, the sections were dehydrated and viewed under the light microscope (400× amplification; Nikon, Japan).

### Histopathology

The fixed kidney tissues were, embedded in paraffin, and sectioned at 5 µm. After dehydration, sections were stained with hematoxylin and eosin (H&E). The histopathological damage was evaluated and recorded using a light microscope (400× amplification; Nikon, Tokyo, Japan).

### Sirius red staining for collagen

The fixed kidney tissues were embedded in paraffin and 5 µm thick sections cut. The sections were stained with the collagen-specific stain, picrosirius red (Sirius Red F3B and a saturated aqueous solution of picric acid) to examine collagen accumulation in kidney tissue. The stained sections were viewed and recorded using a light microscope (400× amplification; Nikon, Japan). Collagen deposition was quantified using the Image J program (NIH) using a single blinded method.

### Assessment of kidney function

Urinary albumin was determined using commercial kit (Nanjing, Jiancheng, Jiangsu, China). Kidney hemodynamic function was evaluated by measures of blood flow velocity and systolic to diastolic pressure ratio of the left renal artery. Mice were anesthetized and placed in the supine position on a heating pad to maintain body temperature at 36–37 °C. Ultrasound kidney function was assessed using a Vevo 770 high-resolution imaging system (Visual Sonics, Canada) equipped with a high-frequency ultrasound probe (RMV-707B). Hair was removed and aqua sonic clear ultrasound gel (Parker Laboratories, Fairfield, NJ) was applied to the lower back to optimize visualization of the renal artery.

### Immunofluorescence of p-STAT3

NRK-52E cells were seeded with a density of 10^4^ cells/well and incubated at 37 °C, 5% CO_2_ for 72 h. The cells were fixed with 4% paraformaldehyde, permeabilized with 0.1% Triton X-100, and blocked with nonspecific antigens with 1% fetal bovine serum. The cells were incubated with anti-p-STAT3 antibody (1:1000, CST, USA) overnight at 4 °C, followed by PE-conjugated secondary antibody (Cat. #SC-3739; 1:200; Santa Cruz, CA, USA) at room temperature for a further 1 h, and the fluorescent nuclear DAPI stain (Invitrogen, Eugene, OR) for 5 min. Then fluorescent images were observed under a confocal laser scanning microscope (400× amplification; Nikon, Japan).

### Immunofluorescent double-staining

For tissue, frozen sections (5 μm thick) of renal tissue were washed three times/5 min with PBS at room temperature. Slides were blocked using 5% bovine serum albumin for 30 min and then incubated overnight at 4 °C with both STAT3 antibody (CST, USA, 1:200) and the antibody of specific marker, anti-Wilms Tumor 1 (Novus Biologicals, Littleton, NBP2-44607, 1:200) or anti-aquaporin 1 (NB600-749, 1:200), respectively. Slides were then correspondingly incubated with two kinds of secondary antibody (TRITC-labeled antibody from Abcam, ab6786, 1:500; Alexa Fluor488-labeled antibody from Abcam, ab150077, 1:500) at 37 °C for 1 h, and washed by PBS for three times/5 min. The cell nuclei were stained with DAPI for 5 min, and all stained sections were viewed by fluorescent confocal microscopy (Nikon, Tokyo, Japan).

### siRNA-induced gene silencing

Silence of gene expression was achieved using siRNA specific target sequences. STAT3 siRNA was obtained from Gene Pharma (Shanghai, China). SiRNA sequence for rat STAT3: sense, 5′-GCAGGAUCUAGAACAGAAATT-3′; and antisense, 5′-UUUCUGUUCUAG AUCCUGCTT-3′. Control siRNA sequence: sense, 5′-UUCUCCGAACGUGUCACGUTT-3′; and antisense, 5′-ACGUGACACGUUCGGAGAATT-3′. NRK-52E cells were transiently transfected with 100 nM siRNA target sequences or negative control siRNA using Lipofectamine 2000 (Invitrogen, CA, USA) according to the manufacturer’s protocol. The transfected cells were then treated with HG for the subsequent studies.

### Statistical analysis

Data were collected from four independent experiments for in vitro studies and 7 mice in each group for in vivo studies, and were presented as mean ± SD. In each experiment, the values obtained were performed with a normality test and the values for statistical analysis (*n* = 4) followed normal distribution. ANOVA and GraphPad Pro (GraphPad, San Diego, CA) were used to analyze the statistical significance between sets of data. Differences were considered to be significant at *p* < 0.05.

## Results

### STAT3 inhibitor, S3I-201, reduces tissue injury and restores normal function in kidneys of diabetic mice

**Fig. 1:**
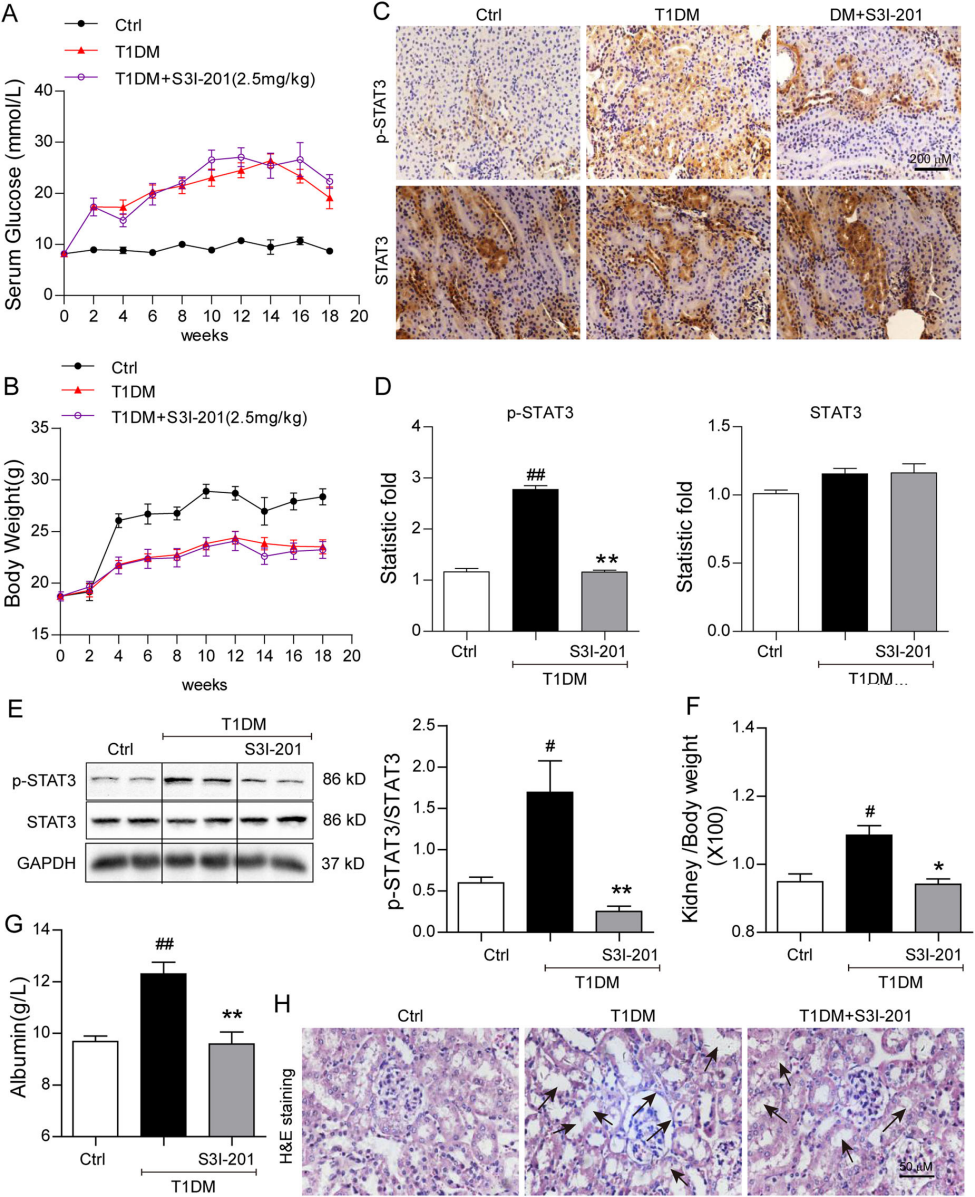
STAT3 inhibitor, S3I-201, reduces tissue injury and restores normal function in kidneys of diabetic mice. Diabetes mellitus was induced with STZ in male C57/BL6 mice, two weeks after which, S3I-201 (2.5 mg/kg) was delivered by IP injection for 16 weeks (Methods), *n* = 7 in each group, Ctrl = citric buffer vehicle control, T1DM = Type 1 diabetes mellitus (DM). Effects of S3I-201 on (**a**) blood glucose (mmol/L) and (**b**) body weight (g) were determined at the indicated weeks. Effects of S3I-201 on STAT3 activation was evaluated by **c** Immunohistochemical detection of phosphorylated STAT3 (brown) and total STAT3; representative images were shown from seven mice per group; **d** Quantification of immunohistochemical images using Image J software; values reported as ratio of the control group, *n* = 7; **e** Western blot analysis of kidney tissue for phosphorylated STAT3, total STAT3, and loading control GAPDH. Shown is representative from seven determinations. Effects of S3I-201 on kidney status were evaluated by **f** ratio of kidney/body weight; **g** urinary albumin (g/L) as index of renal function; **h** Histological image stained with hematoxylin and eosin (H&E) of kidney tissue (arrows indicate structural derangements of tubular epithelia and interstitium), shown is representative from seven determinations, 400× amplification. For **a**, **b**, **d**, **f**, **g**, data are presented as means ± SEM, ^#^*p* < 0.05 and ^##^*p* < 0.01 versus Ctrl; *p < 0.05 and **p < 0.01 versus T1DM

We investigated the protective effects of the selective STAT3 inhibitor, S3I-201 (2.5 mg/kg), on kidney structure and function in the mouse model of STZ-induced DM. Compound S3I-201 is a classic STAT3 inhibitor and shows high selectivity. Previous studies have validated in vitro and in vivo selectivity of S3I-201 for STAT3 versus other STAT and SH2-domaing containing proteins^7^. S3I-201 dose was selected based on study showing inhibition of tyrosine 705 phosphorylation of STAT3 in heart tissues of mice^17^. The diabetic mice presented with hyperglycemia, the blood glucose increasing from < 10 mmol/L to peaking near 30 mmol/L (Fig. 1a). The hyperglycemia was accompanied by significant decreases in body weight gain compared with control mice (Fig. 1b). Treatment with S3I-201 did not alter diabetes-associated hyperglycemia nor the reduced body weight (Fig. 1a, b, respectively).

We next validated the STAT3 inhibition activity of S3I-201 in kidney tissue of diabetic mice. S3I-201 has been shown to block STAT3 phosphorylation at tyrosine 705, thereby inhibiting its DNA-binding activity^7,18^. The kidney tissue of diabetic mice showed increased phosphorylated STAT3 without increasing the total STAT3 as detected by immunohistochemical localization (Fig. 1c, d). As expected, S3I-201 treatment prevented the diabetes-associated increased phosphorylated STAT3 without affecting total STAT3 levels (Fig. 1c, d), western blot analysis of kidney tissues of the diabetic mice corroborated the inhibitory action of S3I-201 on STAT3 phosphorylation (Fig. 1e). The findings indicated that the activated STAT3 occurring in diabetic kidneys was effectively inhibited by S3I-201.

The kidneys of the diabetic mice presented with the expected increased kidney weight/body weight ratio (Fig. 1f) and urinary albumin (Fig. 1g), indicative of kidney dysfunction. The kidney dysfunction in the diabetic mice was accompanied by structural derangements of tubular epithelia, glomerular vascular tufts, and interstitium (Fig. 1h). In the presence of the STAT3 inhibitor, S3I-201, the kidney function and the tubulo-glomerular morphology were comparable to that of control (Fig. 1g, h). Moreover, evaluation of right kidney function in the diabetic mice indicated that both the systolic and diastolic blood flow velocity as well as mean flow velocity (m/s) were significantly decreased, which was accompanied by increased acceleration flow velocity (Table 1). The protective effects of S3I-201 on diabetic kidney structure and function occurred in the absence of lowering blood glucose.

**Table 1:** S3I-201 protects against kidney dysfunction in STZ-induced diabetic mice

	Ctrl (*n* = 7)	DM (*n* = 7)	DM+S3I-201 (*n* = 7)
RKVs (cm/s)	25.888 ± 2.144	14.562 ± 1.284^##^	24.100 ± 2.044**
RKA (m/s^2^)	5.217 ± 0.317	8.473 ± 0.500^##^	6.669 ± 0.492*
RKVd (cm/s)	8.876 ± 0.616	2.802 ± 0.283^##^	5.580 ± 0.835*
RKVm (cm/s)	17.489 ± 0.983	9.469 ± 0.837^##^	14.433 ± 1.710*

*RKVs* systolic flow velocity of right kidney, *RKVd* diastolic flow velocity of right kidney, *RKA (m/s*^*2*^*)* acceleration flow velocity of right kidney, *RKVm* mean flow velocity of right kidney

Data are means ± SEM (*n* = 7; ^#^*p* < 0.05, ^##^*p* < 0.01 versus Ctrl (control); *p < 0.05, **p < 0.01 versus DM group)

### S3I-201 reduces accumulation of collagen and expression of pro-fibrotic signaling molecules in kidney tissue of diabetic mice

**Fig. 2:**
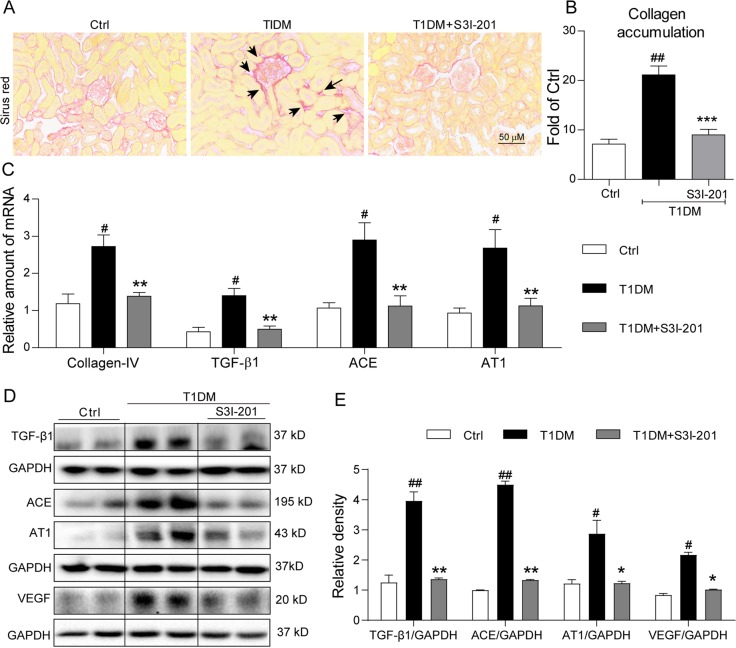
S3I-201 reduces accumulation of collagen and expression of pro-fibrotic signaling molecules in kidney tissue of diabetic mice. The animal treatment and groups were described in Fig. 1 and Methods section; *n* = 7 in each group. **a** Histological depots of collagen fibers (indicated by arrows) as visualized with sirius red staining; shown are representative images from seven mice per group, 400× amplification; **b** Quantification of collagen accumulation from the sirius red stained images using Image J software; values reported as normalized to Ctrl; **c** Kidney tissue collagen IV, TGF-β1, ACE, and AT1 mRNA was determined by quantitative RT-PCR; values normalized to the house-keeping gene, β-actin. **d** Representative Western blot analysis of TGF-β, ACE, AT1, and VEGF in duplicates, GAPDH as loading control. **e** Corresponding densitometric analysis of blots in **e**, calculating from densitometric values of repective bands normalized to loading control GAPDH. For **b**, **c**, and **e**, data are presented as means ± SEM; (*n* = 7; ^#^*p* < 0.05, ^##^*p* < 0.01 versus Ctrl; *p < 0.05, **p < 0.01 versus T1DM group.)

A key histopathological characteristic of DN is tubulo-interstitial fibrosis^2,15^, with collagen IV as the principal extracellular matrix protein produced^19^. Predictably, histochemical detection for collagen using sirius red indicated significant increases of collagen surrounding Bowman’s capsule and within the interstitial compartment (Fig. 2a), and the semi-quantitative analysis indicated a >2-fold increase over control (Fig. 2b). Treatment with the STAT3 inhibitor, S3I-201, effectively reduced the collagen accumulation (Fig. 2a, b). We confirmed with real-time qPCR assay that the increased collagen accumulation in the diabetic mice was predominantly collagen IV, which was accompanied by increased expression of TGF-β1 (Fig. 2c), a potent cytokine that stimulates synthesis of extracellular matrix proteins^19^. In the presence of S3I-201, the increased collagen IV mRNA and TGF-β1 mRNA observed in diabetic kidneys were significantly reduced (Fig. 2c). Western blot analysis of kidney tissue from diabetic mice indicated that TGF-β1 was increased fourfold over control (Fig. 2d, e), while this increase was significantly reversed by S3I-201 administration, supporting STAT3 in promotion tubulo-interstitial fibrosis in DN.

DN is clearly related to an activated renin–angiotensin system (RAS), whereby inhibition of the components of the system [e.g., ACE (angiotensin II generating enzyme) or AT1 (angiotensin II receptor type 1)] can reduce the disease severity^20^. Overexpression of the components of RAS, such as ACE^21^ and AT1^22^, contributes to the pathogenesis of DN. Therefore, we investigated whether STAT3 regulates the expression of ACE and AT1 in our diabetic mouse model. Results indicated that the kidney tissue of diabetic mice presented with increased mRNA levels of ACE and AT1, which were prevented by S3I-201 (Fig. 2c). Western blot analysis of kidney tissue showed similar results at the protein level (Fig. 2d, e). The increased ACE level occurring in kidney tissues of diabetic mice has been reported to induce local production of VEGF in podocytes^23^ and tubular epithelium^24^. VEGF is a pro-angiogenic and endothelial permeability factor implicated in the pathogenesis of DN^1,25^. We then measured the level of VEGF in mouse kidney. Western blot analysis indicated that the kidney tissue of diabetic mice had ~2-fold greater expression of VEGF over control, and the presence of S3I-201 prevented this VEGF increase (Fig. 2d, e). The overall results indicated that S3I-201 was highly effective in limiting increases of collagen accumulation in kidneys of diabetic mice, which also inhibited the upstream signaling components of RAS.

### Silencing STAT3 in kidney attenuated renal injuries in STZ-induced diabetic mice

**Fig. 3:**
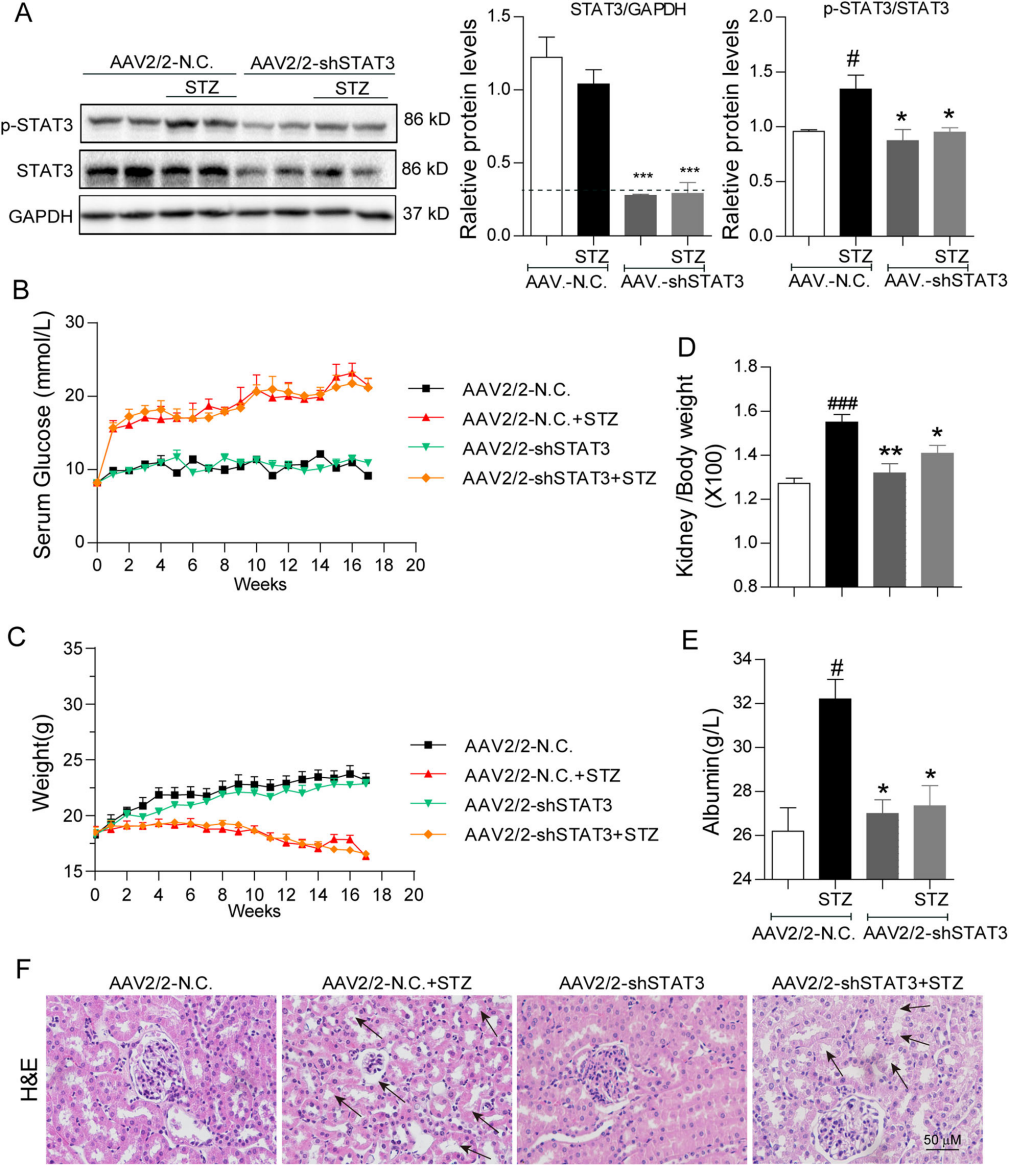
STAT3 knockdown attenuates tissue injuries in kidneys of STZ-induced diabetic mice. The animal treatment and groups were described in Methods section; n = 7 in each group. **a** Effect of AAV2/2-shSTAT3 on STAT3 expression and activation in mouse kidney tissues. Shown are representative western blots of p-STAT3 and STAT3; GAPDH as a loading control; Corresponding densitometric analysis of blots in panel **a** were normalized to loading control GAPDH or STAT3 and reported relative to Ctrl. **b** Blood glucose (mmol/L) was determined at the indicated weeks (from 0 to 17th week). **c** Body weight (g) of mice was recorded at the indicated times. **d** Ratio of kidney/body weight was determined. **e** Urinary albumin (g/L) as an index of renal function was determined. **f** Hematoxylin and eosin (H&E) staining of kidney tissue; shown are representative images from seven mice per group (arrows indicate structural derangements of tubular epithelia and interstitium), 400× amplification. For **a**, **d**, **e**, data are presented as means ± SEM, ^#^*p* < 0.05, ^##^*p* < 0.01, and ^###^*p* < 0.001 versus AAV2/2-NC; *p < 0.05, **p < 0.01, and ***p < 0.001 versus AAV2/2-NC+STZ

**Fig. 4:**
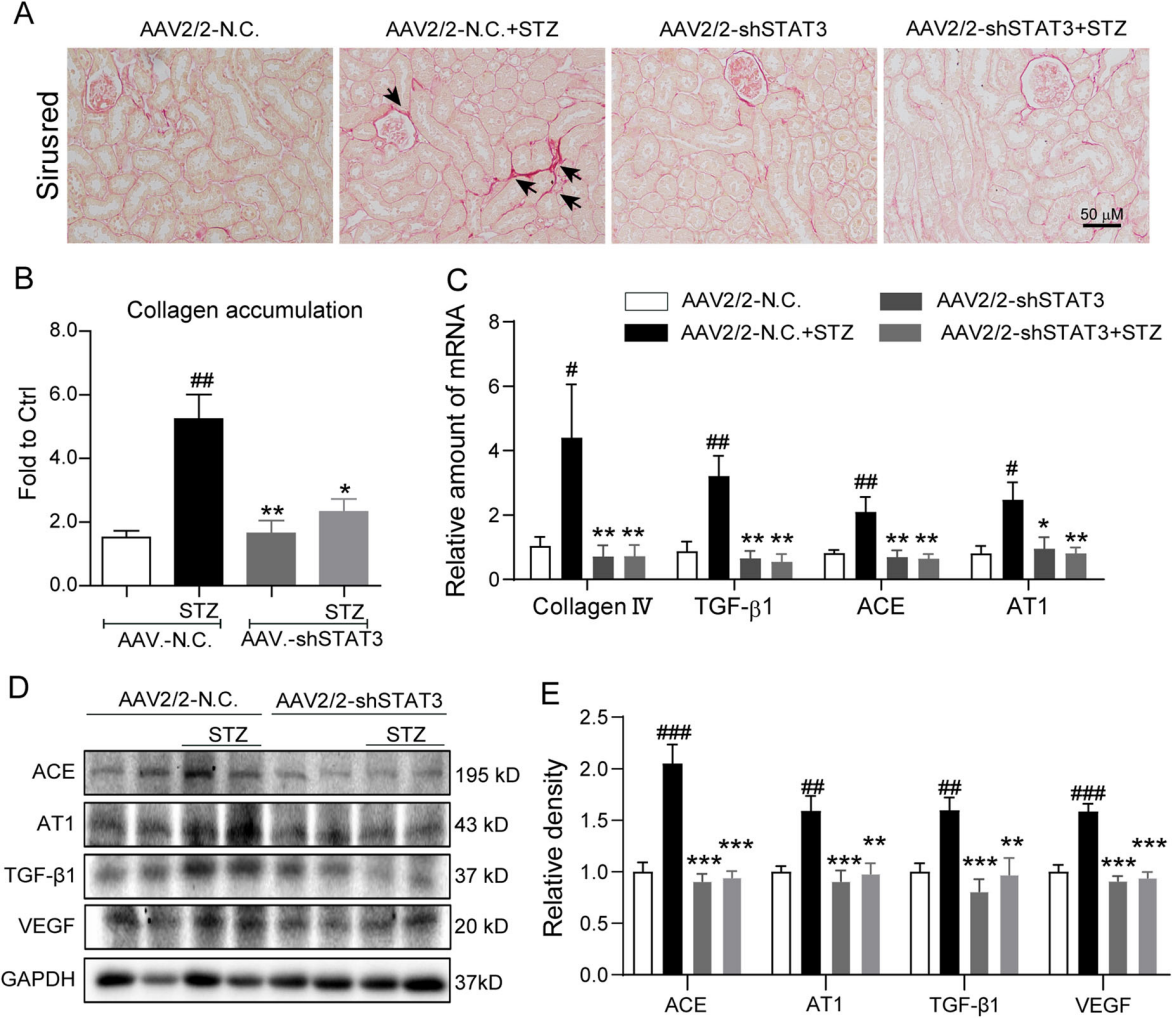
Silencing STAT3 reduces accumulation of collagen and expression of pro-fibrotic signaling molecules in kidney tissue of diabetic mice. The animal treatment and groups were described in Fig. 3 and Methods section; *n* = 7 in each group. **a** Siriu’s red staining of kidney tissue (indicated by arrows); shown are representative images from 7 mice per group, 400× amplification. **b** Quantification of collagen accumulation from the sirius red stained images using Image J software; values reported as normalized to Ctrl; **c** Quantitative real-time PCR determination of collagen IV, TGF-β1, ACE, and AT1 mRNA in mouse kidney tissues; values normalized to house-keeping gene β-actin and reported relative to one of mice in Ctrl group. **d** Effects of STAT3 knockdown on STAT3 activation and related protein expression in mouse kidney tissues. Shown are representative western blots of ACE, AT1, TGF-β, and VEGF; GAPDH as a loading control. **e** Corresponding densitometric analysis of blots in **e** values normalized to loading control GAPDH or STAT3 and reported relative to Ctrl. For **b**, **c**, **e**, data are presented as means ± SEM, ^#^*p* < 0.05, ^##^*p* < 0.01, and ^###^*p* < 0.001 versus AAV2/2-NC; *p < 0.05, **p < 0.01, and ***p < 0.001 versus AAV2/2-NC+STZ

To validate the renal protection of STAT3 inhibition by small molecule, we knocked down STAT3 using adeno-associated virus type 2/mutant 2 expressing STAT3 shRNA (AAV2/2-shSTAT3) in diabetic mice. AAV2/2 has been reported to produce robust expression in renal tissue^26^. We injected AAV2/2-shSTAT3 by tail vein 2 weeks before STZ injection. The results showed that the expression of STAT3 in the kidneys of AAV2/2-shSTAT3 group mice was significantly decreased compared with the AAV2/2-NC group, and the level of renal p-STAT3 was also remarkably reduced in AAV2/2-shSTAT3 mice challenged by STZ (Fig. 3a). Similar with S3I-201, STAT3 knockdown by AAV2/2-shSTAT3 did not affect hyperglycemia and body weight change in STZ-induced diabetic mice (Fig. 3b, c). Silencing STAT3 relieved STZ-induced increases in kidney/body weight ratio and urinary albumin in mice, indicating a functional protection of kidney by STAT3 knockdown (Fig. 3d, e). Histochemical staining showed that treatment with AAV2/2-shSTAT3 remarkably reduced the morphological changes (Fig. 3f) and collagen accumulation (Fig. 4a, b) in diabetic mouse kidney. Further, we measured the levels of related proteins in mouse kidneys. The results showed that STAT3 knockdown prevented STZ-induced gene expression (mRNA and protein levels) of collagen IV, TGF-β1, ACE, AT1, and VEGF in mouse kidney (Fig. 4c–e). Previous study also showed the role of STAT3 in regulation of inflammation and abnormal matrix synthesis at an early stage of DN^12^. Thus, we examined the markers of inflammation and matrix synthesis in AAV-shSTAT3 mouse kidney tissues. The data in the Supplementary Fig. S1 showed that knockdown of STAT3 inhibited diabetes-induced overexpression of TNF-α, IL-1β, MMP-2, and MMP-9. These data in STAT3 gene knockdown validate the reno-protective effects of STAT3 inhibition in diabetic mice.

### STAT3 were mainly expressed in tubular epithelial cells in mouse kidneys

**Fig. 5:**
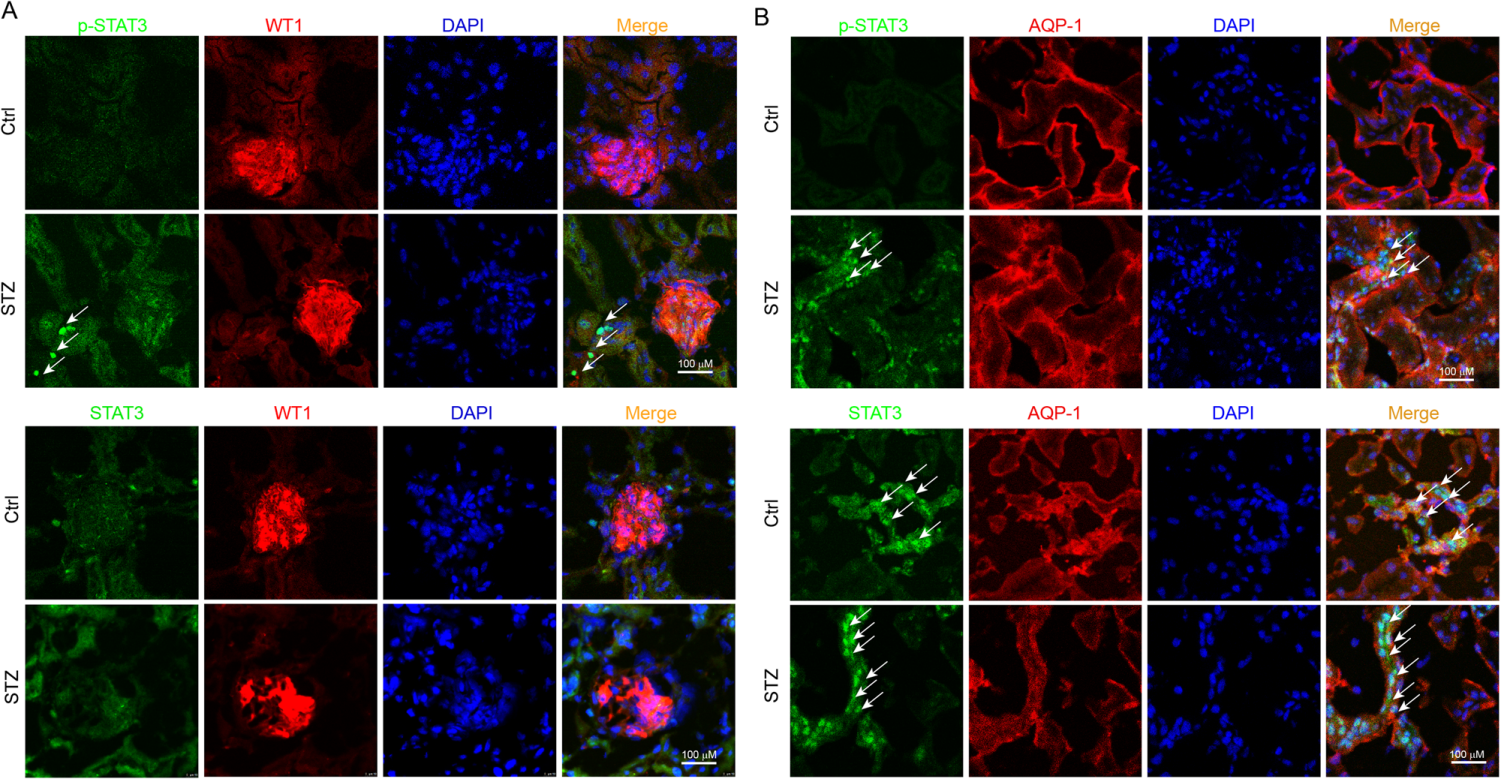
Localization of STAT3 activation during renal lesion development. Kidney tissues (5-μm section) from Control mice and STZ-treated mice (*n* = 7) were processed to immunofluorescent double-staining with antibodies for p-STAT3/STAT3 and WT1 (**a** glomerular cell marker) or APQ1 (**b** renal tubular epithelial cell marker), respectively, as described in Methods section. The arrows point to the nuclear STAT3 protein in the renal tubule cells. Scale bar = 100 μm; 400× amplification

Above data indicated the important role of STAT3 in the pathogenesis of DN. We next evaluated STAT3 localization in kidney tissue. Hyperglycemia initially damages glomeruli and subsequently tubule-interstitial tissues in the development of the DN^11^, indicating the importance of tubular and glomeruli cells. We carried out an immunofluorescent double-staining using p-STAT3/STAT3 antibody and cell-type specific markers to identify p-STAT3/STAT3-positive cell types in mouse kidney. Interestingly, our data showed that both p-STAT3 activation and STAT3 expression were detected mainly in AQP-1-positive tubular epithelial cells, while few in WT1-positive glomeruli cells and podocytes (Fig. 5a, b). In addition, Fig. 5b showed a lot of nuclear STAT3 protein (indicated by arrows) in the tubular cells of STZ-induced diabetic kidney, explaining that hyperglycemia induced STAT3 nuclear translocation and activation, which is consistent with the fact that diabetes increased STAT3 phosphorylation in mouse kidney (Fig. 1e).

### S3I-201 inhibits high glucose-induced pro-fibrotic responses in renal tubular epithelial cells

**Fig. 6:**
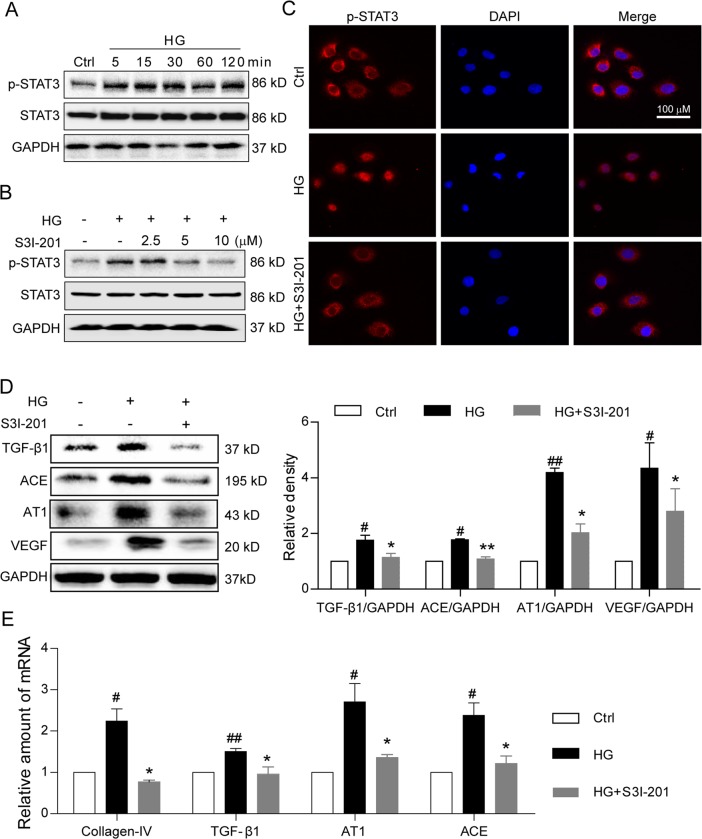
S3I-201 inhibits high glucose-induced STAT3 activation and pro-fibrotic responses in rat renal tubular epithelial cell line. **a** Time-course of STAT3 phosphorylation (Tyr-705) in rat renal tubular epithelial cells (NRK-52E) stimulated with high glucose (HG; 33 mM); shown is representative Western blot analysis, Ctrl = no glucose control, GAPDH = loading control, *n* = 4. **b** Effects of S3I-201 pretreatment on HG-induced STAT3 phosphorylation in NRK-52E cells. Cells were treated with S3I-201 (2.5, 5, and 10 µM) for 1 h, stimulated with HG 30 min, cells collected for Western blot analysis; shown is representative blot from four determinations. **c** For nuclear localization of p-STAT3, S3I-201 pretreated NRK-52E cells were stimulated with HG for 30 min, and immunofluorescence detection of p-STAT3 (red) evaluated (Methods), DAPI = nuclear fluorescence stain (blue), MERGE = overlaid images, *n* = 4. Scale bar = 100 μm; 600 × amplification. **d**, **e** Fibrosis-related proteins and signaling molecules were detected. Rat renal tubular epithelial cells (NRK-52E) were pretreated with S3I-201 (10 µM) for 1 h and stimulated with HG for 24 h. Representative western blot analysis of TGF-β1, ACE, AT1, and VEGF with GAPDH as loading control; corresponding densitometric analysis of blots, values normalized to loading control GAPDH and reported relative to Ctrl (**d**). Quantitative RT-PCR determination of Collagen IV, TGF-β1 AT1, and ACE mRNA, values normalized to house-keeping gene β-actin and reported relative to Ctrl (**e**). Data are represented as the mean ± SEM of four independent experiments; ^#^*p* < 0.05, ^##^*p* < 0.01 versus Ctrl (DMSO control); *p < 0.05, **p < 0.01 versus HG group

We next confirmed the inhibitory action of S3I-201 in a rat renal tubular epithelial cell line, NRK-52E, stimulated with high-concentration glucose (HG, 33 mM). We established the time-course of HG-induced STAT3 activation, showing that HG treatment consecutively induced STAT3 phosphorylation at tyrosine 705 from 15 min to 24 h, but did not increase the STAT3 expression (Fig. 6a and Supplementary Fig. S2). Pretreatment of the NRK-52E cells with S3I-201 reduced the HG-induced STAT3 phosphorylation in a dose-dependent manner, achieving maximal inhibition at 10 µM (Fig. 6b). Moreover, S3I-201’s inhibition of the HG-induced STAT3 phosphorylation corresponded with inhibition of the HG-induced nuclear translocation of p-STAT3 in NRK-52E cells (Fig. 6c). We then evaluated STAT3 activation in NRK-52E cells in contributing to the pro-fibrotic responses observed in the diabetic mouse model. NRK-52E cells were pretreated with S3I-201 (10 µM) for 1 h, followed by stimulation with HG (33 mM) for 24 h. Results indicated HG induced a significant increase over control in the protein expression of TGF-β1, ACE, AT1, and VEGF in NRK-52E cells (Fig. 6d). The pretreatment with S3I-201 prevented HG-induced increases of these proteins (Fig. 6d). Similar results were observed in the mRNA level using real-time qPCR assay (Fig. 6e). We also tested the level of collagen I using real-time qPCR assay. The data in the Supplementary Fig. S3 showed that Collage I has a similar changing profile with collagen IV. Consistent with the results in AAV-shSTAT3 mouse kidney tissues, inhibition of STAT3 by S3I-201 also reduced HG-increased mRNA levels of TNF-α, IL-1β, MMP-2, and MMP-9 (Supplementary Fig. S4). To see the treatment effects of STAT3 inhibitor, we added the examination in NRK-52E cells where S3I-201 was added after HG stimulation. As shown in the Supplementary Fig. S5, treatment with S3I-201 after 1-h HG incubation also prevented the protein expression and mRNA transcription of ACE, AT1, VEGF, and TGF-β1 in NRK-52E cells, indicating STAT3 inhibitor S31-201 has a treatment effect against HG-induced injuries. Overall, the findings indicated that inhibition of STAT3 with S3I-201 effectively reduced the HG-induced activation of pro-fibrotic pathways in renal tubular epithelial cells.

### Knockdown of STAT3 mimics the inhibitory effects of S3I-201 on the high glucose-induced pro-fibrotic responses

**Fig. 7:**
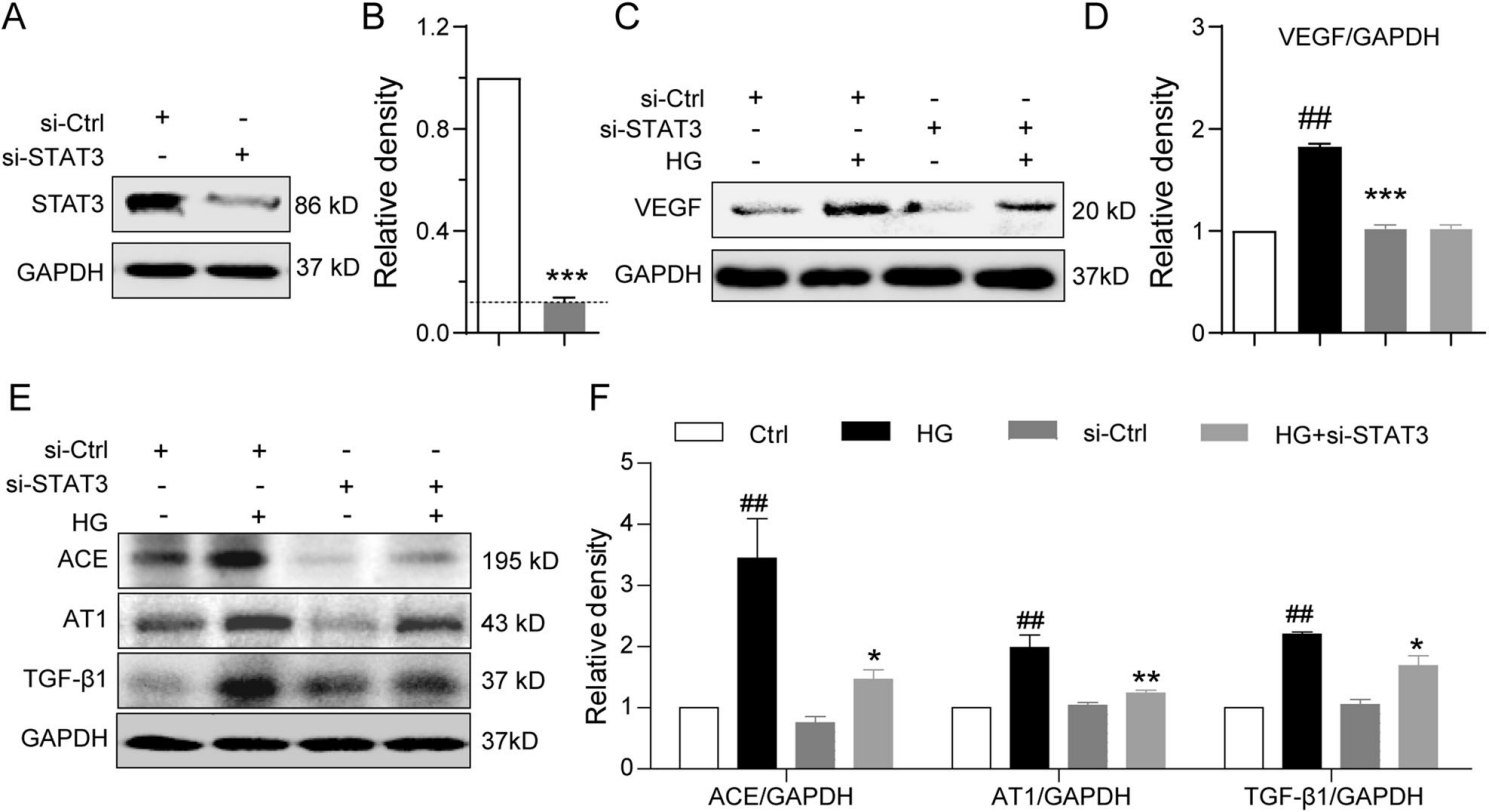
Knockdown of STAT3 prevents high glucose-induced pro-fibrotic responses in rat renal tubular epithelial cells. STAT3 was knockdown using siRNA (si-STAT3) in rat renal tubular epithelial cells (NRK-52E), the cells were stimulated with HG for 24 h for Western blot analysis; GAPDH as loading control, si-Ctrl = scramble sequence. Shown are representative blots indicating **a**, **b** extent of STAT3 knockdown; **c**, **d** VEGF; **e**, **f** ACE, AT1, and TGF-β1. Protein bands were quantified by densitometry, normalized to GAPDH, and reported relative to the vector control group. Data are presented as the mean ± SEM of four independent experiments; ^##^*p* < 0.01 versus Vector control, *p < 0.05, **p < 0.01, versus the HG

To avoid the possible non-specificity of the small-molecule inhibitor S3I-201 on STAT3 inhibition, we employed specific target siRNA sequences to knockdown STAT3 expression in NRK-52E cells. Cells treated STAT3 siRNA showed <10% protein expression compared with the vector control (Fig. 7a, b). The siRNA-mediated STAT3 downregulation prevented the HG-induced increases in protein expression of VEGF, ACE, AT1, and TGF-β1 (Fig. 7c–f). The results of STAT3 downregulation were consistent with those obtained by pharmacological inhibition of STAT3 with S3I-201, providing convincing evidence of a critical role of STAT3 in regulation of the pro-fibrotic activities induced by HG and validating the pharmacological effects of S3I-201.

## Discussion

The central goal of the study was to investigate the pharmacological inhibition of STAT3 as a therapeutic strategy for the treatment of DN, and whether renal tubular epithelial cells contribute to this regulation. Our findings indicated that STAT3 was activated in the in vivo mouse model of STZ-induced DM and in the in vitro HG-stimulated renal tubular epithelial cells. Administration with STAT3 inhibitor, S3I-201, prevents DN in type 1 diabetic mice. Similar results were confirmed using silencing of STAT3 in diabetic mouse kidneys by injections of AAV2 expressing shSTAT3 sequences. This study provided the first evidence that pharmacological inhibition of STAT3 attenuates DN, indicating STAT3 inhibitors may support a new therapeutic strategy for DN.

Our data indicated that STAT3 regulated significant accumulation of collagen in the interstitium along proximal tubules and around glomeruli in kidneys of the diabetic mice, indicative of fibrotic activities. The pro-fibrotic activities in the diabetic mouse kidney were accompanied by kidney dysfunction, as indicated by increased proteinuria as well as impaired renal hemodynamics, indicating that our STZ-induced diabetic model presented with progressive DN. It is slightly controversial if STZ-induced diabetes causes kidney fibrosis in C57BL/6 mice. Although Kitada et al. observed no tubulo-interstitial fibrosis in STZ-induced C57BL/6 mice^27^, a lot of papers from our group^28,29^ and other groups^30,31^ reported kidney fibrosis in STZ-induced C57BL/6 mice. Therefore, we consider that the induction of kidney fibrosis may depend on the duration of hyperglycemia. In both animal experiments of this study, the kidney fibrosis was clearly observed. The pro-fibrotic activities and kidney dysfunction were tightly coupled since STAT3 blockade with S3I-201 prevented both activities. These findings are further support of the important role of STAT3 in the pathogenesis of DN as reported by others^6,9,12^.

We found that STAT3 was expressed in mouse tubular epithelial cells and HG stimulated renal tubular epithelial cells to increase expression of collagen IV and the fibrosis-related signaling molecules, i.e., TGF-β1. TGF-β is a potent cytokine known to increase synthesis of extracellular matrix proteins, such as collagen IV^19^. The finding suggests that tubular epithelial cells can be an active participant of the pro-fibrotic tissue milieu, contributing to extracellular matrix (ECM) deposition and driving the progression of DN. Moreover, the significant accumulation of collagen in the interstitium along proximal tubules and around glomeruli of the diabetic kidney would be consistent with local production of collagen and other ECM proteins. Consistent with this possibility is the observation that the STAT3 inhibition or knockdown prevented the pro-fibrotic activities in the HG-stimulated renal epithelial cells as well as the increased tissue accumulation of collagen. To-date, the role of STAT3 in DN has been reported only in mesangial cells^9,10^, and little is known of potential involvement by other kidney cell populations^4^. The results from this study support a new finding that the STAT3 signaling appears to be a mechanism by which renal epithelial cells also regulate pro-fibrotic activities, contributing to the overall progression of DN. Therefore, STAT3 in renal tubular epithelial cells contributes to the pathogenic mechanism of DN. This study may have a limitation our current data cannot exclude the role of STAT3 in other cell types in DN progression. Future studies should be performed using the primary TECs and tubular-specific STAT3-knockout mice. In addition, we did not see the distribution of p-STAT3/STAT3 in other organs (spleen, liver, peripheral blood, etc...). Such information could be useful for further preclinical and clinical study. Although our in vitro data using NRK-52E and in vivo data using AAV2/2-shSTAT3 validated the role of renal STAT3 in diabetic nephropathy, the inhibition of STAT3 in other organs by globe treatment with S3I-201 may also contribute to its renal protection.

Our results indicated that activated STAT3 in the kidney of diabetic mice signaled the activation of the RAS system, specifically ACE and AT1, suggesting elevated Ang II generation as well as heightened AT1 activity. An increased Ang II generation is critically important in the pathogenesis of DN, impacting on both hemodynamic effects and cellular activities^32^. We also observed that the HG-induced increase of ACE and AT1 in renal tubular epithelial cells was also dependent on STAT3 activation. This finding suggests that the renal tubular epithelial cells likely contribute to the increased levels of Ang II in the progression of DN. The importance of RAS activation in DN is underscored by the number of investigations indicating that the development and progression of DN can be limited by normalizing blood pressure through blockade of ACE and AT1^33^.

Increasing evidence supports a direct role of VEGF in the pathogenesis of DN, contributing to glomerular hyperpermeability, macrophage infiltration, cellular growth and proliferation, and mesangial expansion^34^. We found that STAT3 signaled the increased expression of VEGF in both the in vivo kidney of diabetic mice as well as in HG-stimulated renal tubular epithelial cells. This is consistent with the report that VEGF is upregulated in DN in experimental models^35^ and in renal biopsies from human diabetic subjects^36^. We suspect that the increased VEGF expression in the diabetic mice or HG-stimulated renal tubular epithelial cells was attributed to direct STAT3-dependent transcription of the gene, since STAT3-dependent genes include chemokines, growth factors, and extracellular matrix proteins^8^.

In summary, activated STAT3 occurred in kidney tissue of STZ-induced diabetic mouse model and in the in vitro HG-stimulated renal tubular epithelial cells, of which, the STAT3 inhibitor, S3I-201, abrogated the STAT3 activation in both experimental models. In addition, genetic knockdown of STAT3 using AAV2/2-shSTAT3 attenuated DN and we also observed the same results in HG-induced NRK-52E cells treated with S3I-201 or siRNA. Moreover, renal tubular epithelial cells contributed to the STAT3 pathogenic mechanism of DN, likely functioning as a local cellular source of ECM deposition. The results support the new therapeutic strategy for DN by using small-molecule STAT3 inhibitors. However, a limitation of this study is that we only used mouse model of STZ-induced type 1 diabetic nephropathy. Diabetic nephropathy happens in many patients with type 2 diabetes and a progression of diabetic nephropathy may be better reflected in type 2 diabetic mice. Future study on the renal protective effect of S3I-201 should be performed in type 2 diabetic mice to further validate the therapeutic strategy for DN.

## Supplementary information

Supplementary Figure Legends

Supplementary Figure S1

Supplementary Figure S2

Supplementary Figure S3

Supplementary Figure S4

Supplementary Figure S5

DECLARATION OF CONTRIBUTIONS TO ARTICLE
