# Supplementary material for: Inhibition of STAT3 in tubular epithelial cells prevents kidney fibrosis and nephropathy in STZ-induced diabetic mice
Source: Cell Death Dis. 2019 Nov 7;10(11):848. doi: 10.1038/s41419-019-2085-0 (PMC6838321; doi:10.1038/s41419-019-2085-0)
Supplement: Supplementary file 1 — Supplementary Figure Legends [file 41419_2019_2085_MOESM1_ESM.docx]

***Supplementary information***

**Inhibition of STAT3 in tubular epithelial cells prevents kidney fibrosis and nephropathy in STZ-induced diabetic mice**

Chao Zheng^1,2,#,^*, Lan Huang^1,#^, Wu Luo^1,2,4,#^, Weihui Yu^3^, Xueting Hu^1^, Xinfu Guan^4^, Yan Cai^4^, Chunpeng Zou^5^, Haimin Yin^1,2^, Zheng Xu^1^, Yi Wang^1, 4^*

^1^ Chemical Biology Research Center, School of Pharmaceutical Science, Wenzhou Medical University, Wenzhou, Zhejiang 325035, China

^2^ Department of Endocrinology, the Second Affiliated Hospital, Wenzhou Medical University, Wenzhou, Zhejiang 325000, China

^3^ Department of Endocrinology, the First Affiliated Hospital, Wenzhou Medical University, Wenzhou, Zhejiang 325035, China

^4^ The Affiliated Cangnan Hospital, Wenzhou Medical University, Wenzhou, Zhejiang 325400, China

^5^ Department of Ultrasonography, the Second Affiliated Hospital, Wenzhou Medical University 325000, Wenzhou, Zhejiang, China

**Supplementary Table S1. Primer sequences for real-time quantitative PCR**

| Gene | Species | Forward primer | Reverse primer |
| --- | --- | --- | --- |
| ACE | RAT | CACCGGCAAGGTCTGCTT | CTTGGCATAGTTTCGTGAGGAA |
| AT1 | RAT | GGAAACAGCITGGTGGTG | GCACAATCGCCATAATTATCC |
| TGF-β1 | RAT | GCAACAACGCAATCTATGAC | CCTGTATTCCGTCTCCTT |
| Collagen IV | RAT | CG*GGATTC*ATGGGACCGCCAGGTTTA | TCCC*CTCGAG*TCATGACTTTGAATA |
| TNF-α | RAT | TACTCCCAGGTTCTCTTCAAGG | GGAGGCTGACTTTCTCCTGGTA |
| IL-1β | RAT | CACCTCTCAAGCAGAGCACAG | GGGTTCCATGGTGAAGTCAAC |
| MMP2 | RAT | AGAGGATACCCCAAGCCACT | AATAGCTGTGACCACCACCC |
| MMP9 | RAT | CTGTCCAGACCAAGGGTACAG | CAGGTTTAGAGCCACGACCA |
| Collagen I | RAT | TTTCCCCCAACCCTGGAAAC | CAGTGGGCAGAAAGGGACTT |
| β-actin | RAT | ATCGTGGGCCGCCCTAGGCACC | CTCTTTAATGTCACGCACGATTTC |
| ACE | mouse | CGCACGACACCAACATCAC | GCCAAATGGACTCATACAACTCC |
| AT1 | mouse | AACAGCTTGGTGGTGATCGTC | CATAGCGGTATAGACAGCCCA |
| TGF-β1 | mouse | TGGAGCAACATGTGGAACTC | GTCAGCAGCCGGTTACCA |
| Collagen IV | mouse | CAAGGACCGGTTTATTTGGC | ATTCCCTGCGAAGAACACAGC |
| MMP2 | mouse | CAAGTTCCCCGGCGATGTC | TTCTGGTCAAGGTCACCTGTC |
| MMP9 | mouse | CTGGACAGCCAGACACTAAAG | CTCGCGGCAAGTCTTCAGAG |
| TNF-α | mouse | TGATCCGCGACGTGGAA | ACCGCCTGGAGTTCTGGAA |
| β-actin | mouse | CCGTGAAAAGATGACCCAGA | TACGACCAGAGGCATACAG |

**Supplementary figure legends**

**Supplementary Figure S1. STAT3 knockdown attenuates inflammation and matrix synthesis in kidneys of STZ-induced diabetic mice.** The animal treatment and groups were described in Methods; n=7 in each group. Quantitative real-time PCR determination of TNF-α **(A)**, IL-1β **(B)**, MMP-2 **(C)**, and MMP-9 **(D)** mRNA in mouse kidney tissues; values normalized to house-keeping gene β-actin and reported relative to one of mice in Ctrl group. Data are presented as means±SEM, ^#^p<0.05 and ^##^p<0.01 versus AAV2/2-NC; *p<0.05 and **p<0.01 versus AAV2/2-NC+STZ.

**Supplementary Figure S2.** Time-course of STAT3 phosphorylation (Tyr-705) in rat renal tubular epithelial cells (NRK-52E) stimulated with high glucose (HG; 33 mM). (**A**) Cultured NRK-52E cells were stimulated by HG (33mM) for indicated times. Representative western blot analysis of p-STAT3/STAT3 with GAPDH as loading control. **(B-C)** Corresponding densitometric quantification of blots in panel A, values normalized to loading control GAPDH and reported relative to Ctrl.

**Supplementary Figure S3. S3I-201 reduced HG-increased mRNA level of collagen I in NRK-52E cells.** Rat renal tubular epithelial cells (NRK-52E) were pre-treated with S3I-201 (10 µM) for 1h and then stimulated with HG (33mM) for 24h. Quantitative RT-PCR determination of collagen I mRNA, values normalized to house-keeping gene β-actin and reported relative to Ctrl. Data are represented as the mean±SEM of 4 independent experiments; ##p<0.01 versus Ctrl (DMSO control); **p<0.01 versus HG group.

**Supplementary Figure S4. S3I-201 reduced HG-increased mRNA levels of TNF-α, IL-1β, MMP-2, and MMP-9 in NRK-52E cells.** Rat renal tubular epithelial cells (NRK-52E) were pre-treated with S3I-201 (10 µM) for 1h and then stimulated with HG (33mM) for 24h. Quantitative RT-PCR determination of TNF-α **(A)**, IL-1β **(B)**, MMP-2 **(C)**, and MMP-9 **(D)** mRNA, values normalized to house-keeping gene β-actin and reported relative to Ctrl. Data are represented as the mean±SEM of 4 independent experiments; #p<0.05, ##p<0.01, and ###p<0.001 versus Ctrl (DMSO control); *p<0.05, **p<0.01, and ***p<0.001 versus HG group.

**Supplementary Figure S5.** **Post-treatment of S3I-201 inhibits HG-induced pro-fibrotic responses in** NRK-52E **cells.** Rat renal tubular epithelial cells (NRK-52E) were stimulated with HG (33mM) for 1h and then treated with S3I-201 (10 µM) for 23 h. Fibrosis-related proteins and signaling molecules were detected by Western blot analysis and real-time qPCR from 4 determinations. **(A)** Representative western blot analysis of TGF-β1, ACE, AT1 and VEGF with GAPDH as loading control. **(B-E)** Corresponding densitometric quantification of blots in panel A, values normalized to loading control GAPDH and reported relative to Ctrl. **(F)** Quantitative RT-PCR determination of Collagen IV, TGF-β1 AT1, and ACE mRNA, values normalized to house-keeping gene β-actin and reported relative to Ctrl. Data are represented as the mean±SEM of 4 independent experiments; #p<0.05, ##p<0.01 versus Ctrl (DMSO control); *p<0.05, **p<0.01 versus HG group.
