# Supplementary figures and images for: Inhibition of STAT3 in tubular epithelial cells prevents kidney fibrosis and nephropathy in STZ-induced diabetic mice
Source: Cell Death Dis. 2019 Nov 7;10(11):848. doi: 10.1038/s41419-019-2085-0 (PMC6838321; doi:10.1038/s41419-019-2085-0)

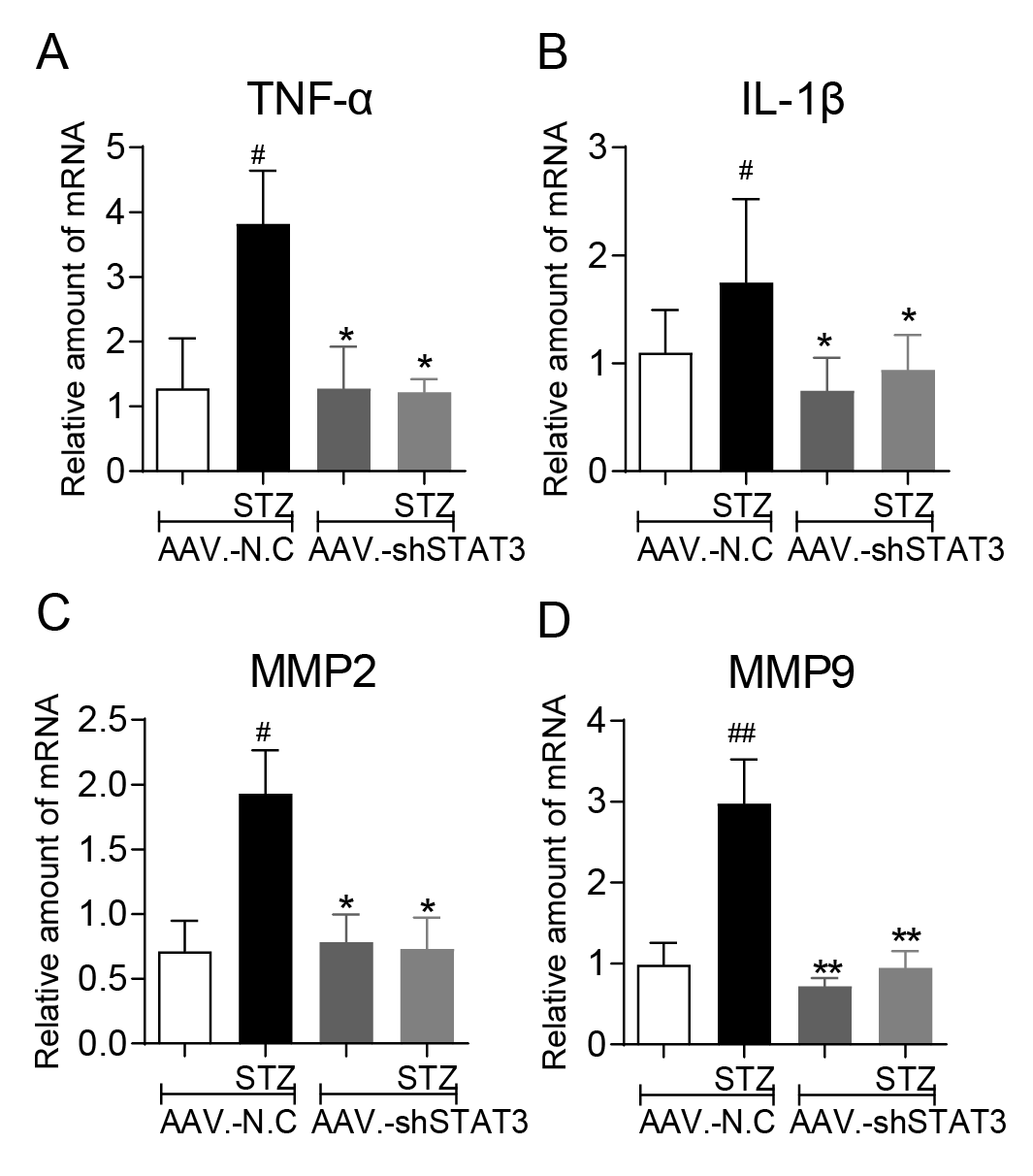

Supplement: Supplementary file 2 — Supplementary Figure S1 [file 41419_2019_2085_MOESM2_ESM.tif]

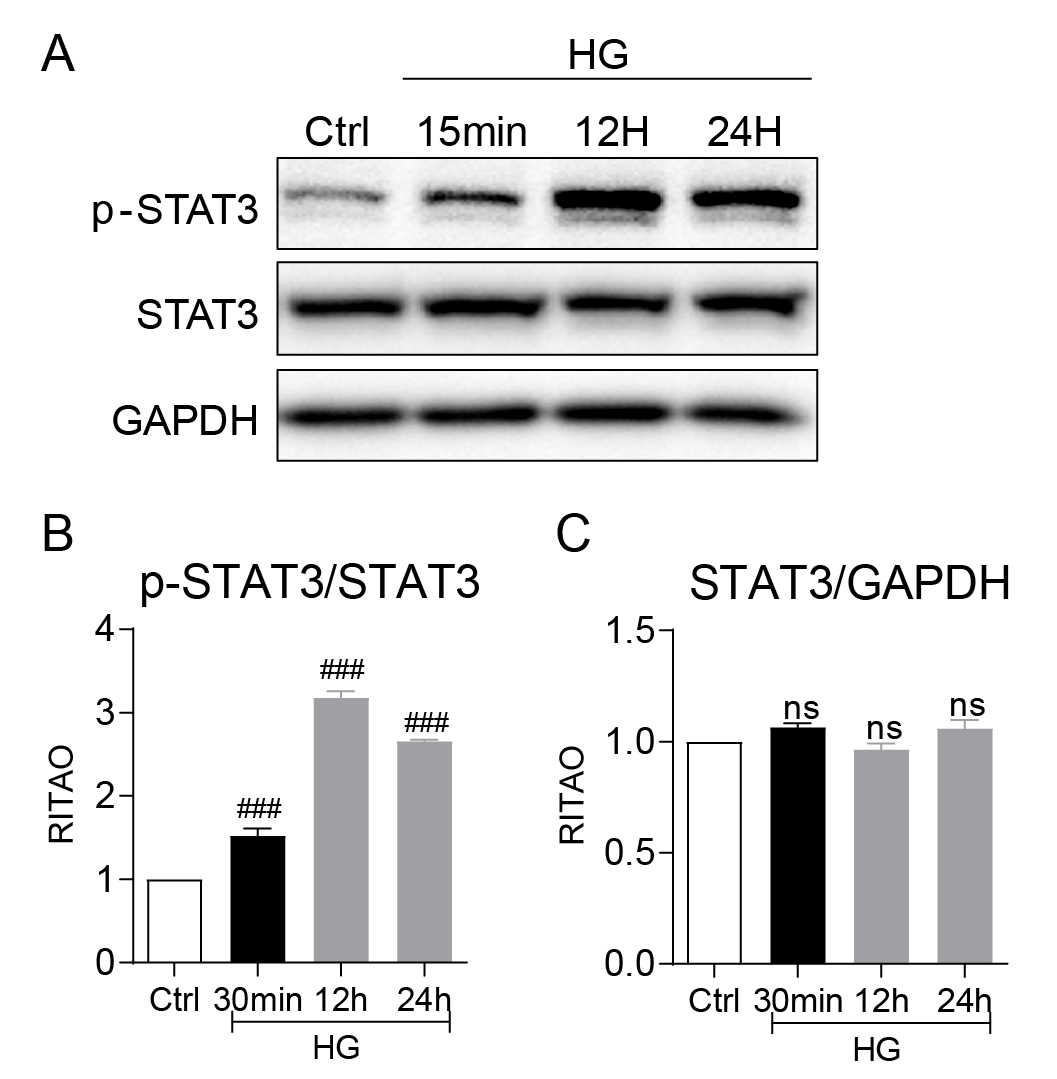

Supplement: Supplementary file 3 — Supplementary Figure S2 [file 41419_2019_2085_MOESM3_ESM.tif]

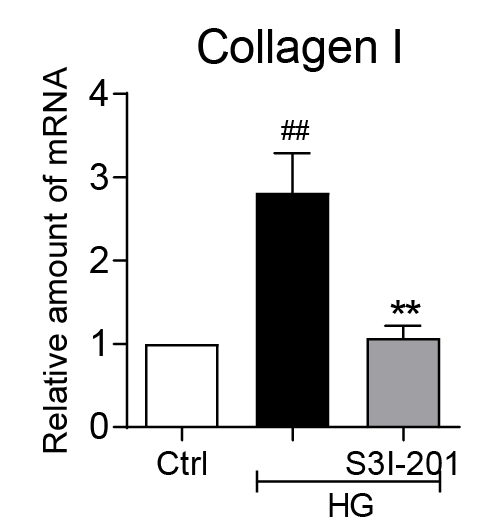

Supplement: Supplementary file 4 — Supplementary Figure S3 [file 41419_2019_2085_MOESM4_ESM.tif]

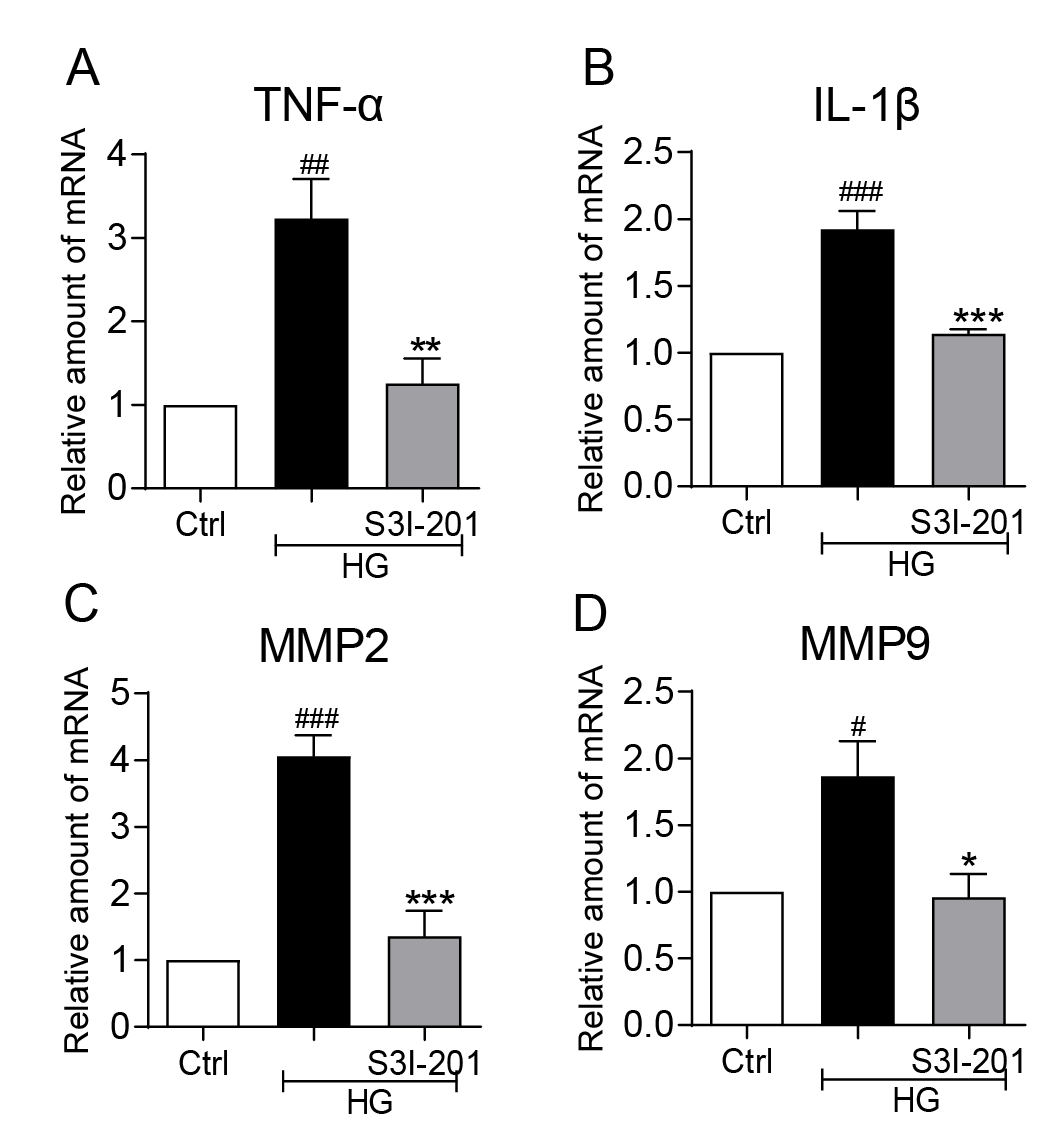

Supplement: Supplementary file 5 — Supplementary Figure S4 [file 41419_2019_2085_MOESM5_ESM.tif]

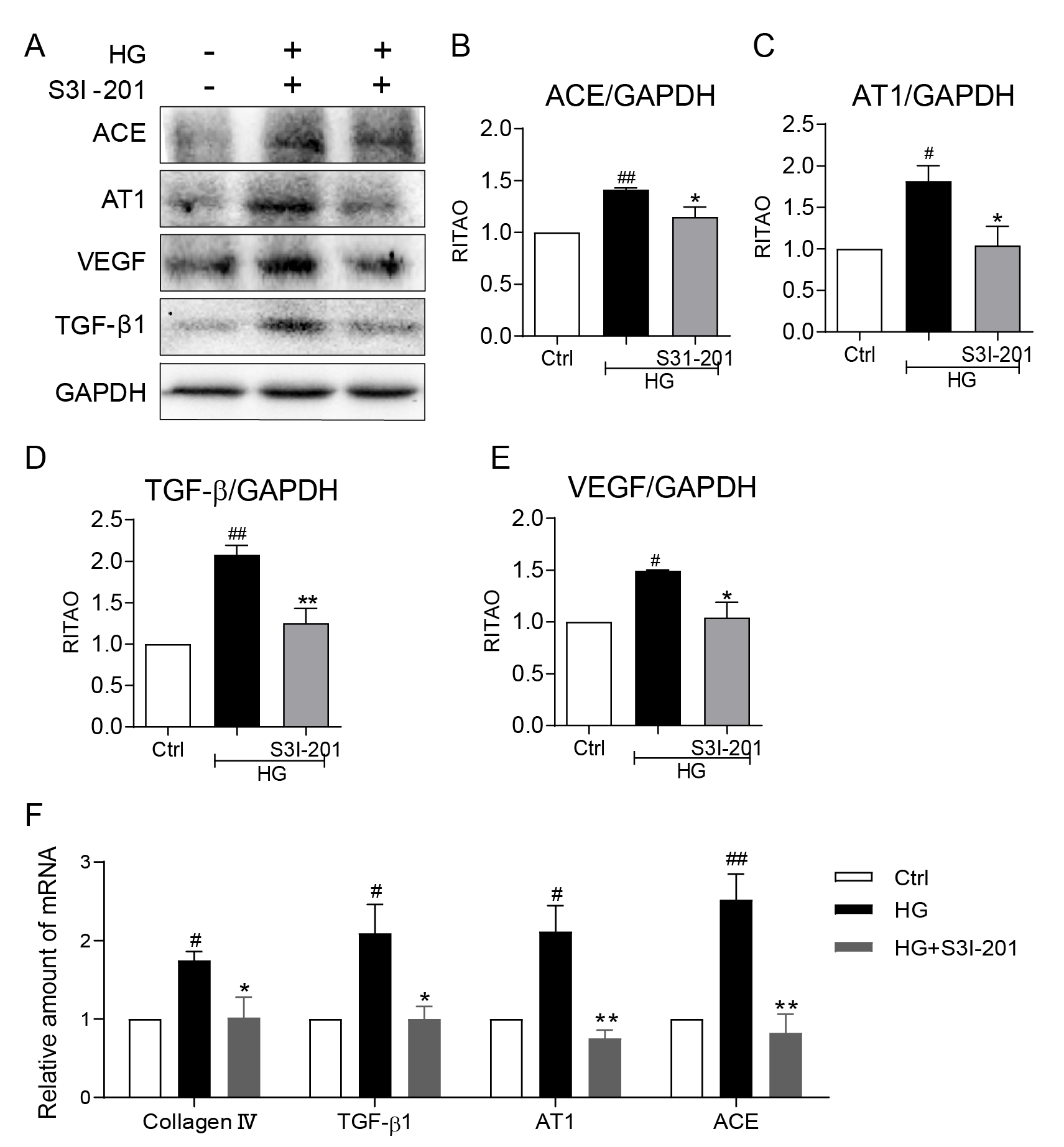

Supplement: Supplementary file 6 — Supplementary Figure S5 [file 41419_2019_2085_MOESM6_ESM.tif]
