# Supplementary material for: Inhibition of STAT3 in tubular epithelial cells prevents kidney fibrosis and nephropathy in STZ-induced diabetic mice
Source: Cell Death Dis. 2019 Nov 7;10(11):848. doi: 10.1038/s41419-019-2085-0 (PMC6838321; doi:10.1038/s41419-019-2085-0)
Supplement: Supplementary file 7 — DECLARATION OF CONTRIBUTIONS TO ARTICLE [file 41419_2019_2085_MOESM7_ESM.pdf]

DECLARATION OF CONTRIBUTIONS TO ARTICLE

ADMC

Manuscript Number: CDDIS-19-1276RR

Journal Name: *Cell Death & Disease* (the 'Journal')

Proposed Title of the Contribution: Author contributions (the 'Contribution)

Author(s): Yi Wang (the 'Authors')

For all *CDDis* articles, each person named as an author in the published version must be able to show he or she has contributed substantially to the article.

Authorship credit should be based on 1) substantial contributions to conception and design, acquisition of data, or analysis and interpretation of data; 2) drafting the article or revising it critically for important intellectual content; and 3) final approval of the version to be published. Authors should meet conditions 1, 2 and 3.

Any person who cannot be shown to have made a substantial contribution to the article cannot be listed as an author in the final version. The name of any person who is deemed to have made a minor contribution can, however, appear in the Acknowledgments section of the article.

Please complete the table below to indicate the contributions of all named authors to the manuscript.

Author Full Name:	Specification of Contribution to the Manuscript:
Yi Wang	designed the study,interpreted the results,wrote the manuscript and analyzed data before publication
Chao Zheng	designed the study,interpreted the resultsand analyzed data before publication
Lan Huang	carried out experiments and collected data
Wu Luo	carried out experiments and collected data,wrote the manuscript and analyzed data before publication
Weihui Yu	carried out experiments and collected data
Xueting Hu	carried out experiments and collected data
Xinfu Guan	designed the study
Yan Cai	designed the study and interpreted the results
Chunpeng Zou	designed the study and interpreted the results
Haimin Yin	carried out experiments and collected data
Zheng Xu	carried out experiments and collected data
Guang Liang	designed the study,interpreted the results,wrote the manuscript and analyzed data before publication

Please complete the table below to indicate the contributions of all named authors to the figures.

Figure 1:

Lan Huang, Zheng Xu, Haimin Yin, Xueting Hu, Yi Wang,Chao Zheng,Guang Liang

Figure 2:

Lan Huang, Zheng Xu, Haimin Yin, Xueting Hu, Yi Wang,Chao Zheng,Guang Liang

Figure 3:

Lan Huang, Zheng Xu, Xueting Hu, Yi Wang, Wu Luo,Guang Liang

Figure 4:

Lan Huang, Zheng Xu, Xueting Hu, Yi Wang, Wu Luo,Guang Liang

Figure 5:

Lan Huang, Zheng Xu, Xueting Hu, Yi Wang, Wu Luo,Guang Liang

Figure 6:

Lan Huang, Zheng Xu, Haimin Yin, Xueting Hu, Yi Wang,Chao Zheng,Guang Liang

Signed for and on behalf of the Author(s):	Print Name:	Date:
Yi Wang	Yi Wang	10/17/2019
